# That Instantaneous Light

**Published:** 1883-06

**Authors:** 


### THAT INSTANTANEOUS LIGHT.

This plausible instrument like many others of the same character will doubtless be very profitable to the venders. It comes to the benighted sections of the country all the way from Boston town, and is nothing more than a cheap galvanic arrangement in a box to ignite instantaneously a common kerosene lamp. The dupe that buys one will find on trial that he has just paid five dollars for a very unwieldly match with which to light a kerosene lamp.

